# Imaging mitotic processes in three dimensions with lattice light-sheet microscopy

**DOI:** 10.1007/s10577-021-09656-3

**Published:** 2021-03-11

**Authors:** Yuko Mimori-Kiyosue

**Affiliations:** Laboratory for Molecular and Cellular Dynamics, RIKEN Center for Biosystems Dynamics Research, 2-2-3 Minatojima-minamimachi, Chuo-ku, Kobe, 650-0047 Japan

**Keywords:** Lattice light-sheet microscopy (LLSM), live imaging, mitosis, chromosome, microtubules, EB1, organelles, cell shape

## Abstract

There are few technologies that can capture mitotic processes occurring in three-dimensional space with the desired spatiotemporal resolution. Due to such technical limitations, our understanding of mitosis, which has been studied since the early 1880s, is still incomplete with regard to mitotic processes and their regulatory mechanisms at a molecular level. A recently developed high-resolution type of light-sheet microscopy, lattice light-sheet microscopy (LLSM), has achieved unprecedented spatiotemporal resolution scans of intracellular spaces at the whole-cell level. This technology enables experiments that were not possible before (e.g., tracking of growth of every spindle microtubule end and discrimination of individual chromosomes in living cells), thus providing a new avenue for the analysis of mitotic processes. Herein, principles of LLSM technology are introduced, as well as experimental techniques that became possible with LLSM. In addition, issues remaining to be solved for use of this technology in mitosis research, big image data problems, are presented to help guide mitosis research into a new era.

## Introduction

Capturing dynamic processes of mitosis at the whole-cell level is necessary to understand how cells coordinate various events, such as attachment of spindle microtubules to kinetochores, interactions between astral microtubules and the cell cortex, and chromosome congression and separation. However, insufficient performance of conventional imaging techniques in terms of resolution and speed has crucially limited what can be observed (Liu et al. 2015).

In the seventeenth century, Hooke (Hooke 1665), van Leeuwenhoek (Leeuwenhoek 1682), and others discovered the “cell” using a newly developed device, the “microscope.” In the early 1880s, the pioneering studies of mitosis were started by German anatomist Walther Flemming (Flemming 1882; Mitchison and Salmon 2001; Paweletz 2001), who coined the term mitosis (*thread* in Greek) from the shape of mitotic chromosomes. More than half a century later, the first observation of a spindle apparatus within a living cell was made by W. J. Schmidt and Sinya Inoué, who used polarizing light microscopy (Inoué 1953; Schmidt 1939).

**Fig. 1:**
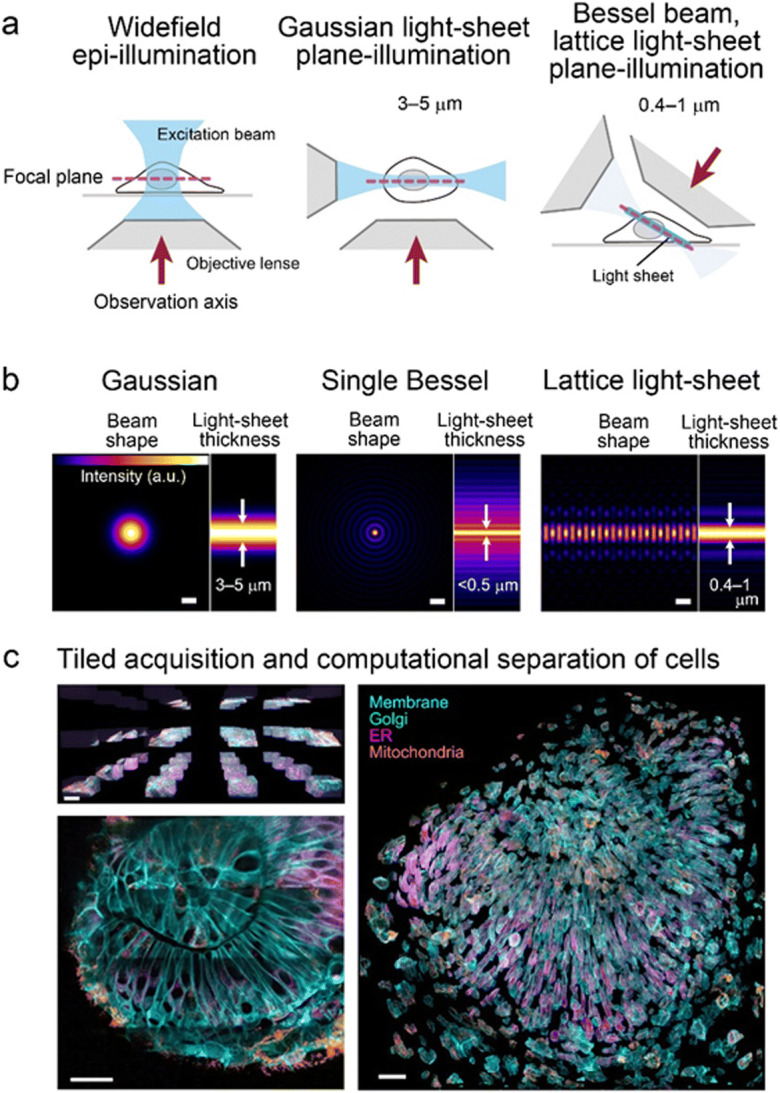
Schematic comparisons of the optical sectioning power and beam shapes used in different illumination techniques, and methods for large volume imaging with LLSM. **a** Optical sectioning power of wide-field epi-illumination, Gaussian light-sheet, and Bessel beam/lattice light-sheet plane illumination techniques. Thickness of irradiation areas are shown in light blue. **b** Beam shapes of three different light-sheet techniques. Light-sheet thickness is shown on the right of each panel. Left: A Gaussian beam is used in traditional laser-scanning light-sheet microscopy (Keller et al. 2008). To create a virtual light-sheet, the beam is swept rapidly, providing time-averaged uniform illumination. Middle: Scanned Bessel beams in conjunction with structured illumination and/or two-photon excitation to create thinner light sheets (<0.5 μm) (Planchon et al. 2011). Right: Multiple Bessel beams arranged in a lattice pattern increases the sample scanning speed (Chen et al. 2014). Scale bars: 1 μm. Adapted from Ref. Chen et al. 2014. **c** Tiled acquisition and computational separation of each cell to image large volumes with LLSM. Upper left: Tiled array across the eye of a developing Zebrafish embryo (upper left). Scale bar: 30 μm. Lower left: Multiple subvolume tiles were stitched to generate the large volume image. Scale bar: 20 μm. Right: Computationally separated cells across the eye in a virtual space to visualize individual cells. Pseudocoloring of the image is indicated by the color of the characters. Scale bar, 30 μm. Adapted from Ref. Liu et al. 2018 under the Copyright (5004591427363)

In this long history of cell biology of mitosis, however, whole-cell three-dimensional (3D) live imaging with sufficient spatiotemporal resolution to discriminate nanoscale-sized mitotic machineries and completely track their movements has never been achieved. The problem becomes particularly relevant when attempting to image subcellular dynamics using fluorescence microscopy (e.g. by introducing green fluorescent protein (GFP) technology (Tsien 1998; Zimmer 2002)), which enables systematic imaging of protein localization in living cells, as well as the structure and function of living tissues. Unfortunately, traditional imaging tools such as confocal microscopy, which uses an epi-illumination configuration that exposes the entire sample thickness to illuminating radiation (Fig. 1a, left), induce out-of-focus background signals that reduce signal-to-noise ratio and increase phototoxicity (Icha et al. 2017; Laissue et al. 2017) and fluorescence photobleaching, and are often too slow to study minute subcellular processes in 3D detail across the entire cell volume. In addition, resolution in an epi-illumination configuration is substantially worse in the axial direction than the lateral plane (Gustafsson and Sedat 1999), which hampers precise measurement of 3D information.

Unlike popular epi-illumination, plane illumination microscopy limits excitation to the vicinity of the focal plane (Fig. 1a, middle and right), which provides effective optical sectioning and high-speed acquisition while minimizing out-of-focus background and photobleaching/phototoxicity (Huisken and Stainier 2009; Mertz 2011; Pampaloni et al. 2007; Planchon et al. 2011; Wan et al. 2019). Conventional light-sheet microscopes using Gaussian beams that are several microns thick were optimized for large specimens, such as small embryos and tissues (Fig. 1b, left). Recently, LLSM using an ultrathin (~ 0.4–1 μm) light sheet was developed (Fig. 1b, right), yielding superior signal-to-background ratios, reduced photobleaching/phototoxicity, and ultrafast plane-wise imaging rates that enable whole-cell 3D scanning, often at subsecond intervals (Chen et al. 2014).

In this review, technical principles of LLSM and examples of its application are introduced to demonstrate how LLSM surpasses conventional microscopic technologies. In addition, critical challenges in image and data analysis that require solving to innovate future mitosis research using LLSM technology are discussed.

## Principles and advantages of LLSM

LLSM is an advanced type of light-sheet microscopy (Chen et al. 2014; Wan et al. 2019). The advantage of light-sheet illumination is that it makes 3D live-imaging approaches to collect precise and reliable 3D information feasible. Unlike frequently used point-scanning techniques, such as confocal and two-photon microscopy, light-sheet microscopes illuminate specimens as a plane with a thin laser beam, while simultaneously imaging this plane with a camera in one shot (Fig. 1a). This approach achieves a combination of crucial properties for live imaging: high imaging speed and signal-to-noise ratio, and low energy load on the specimen, which minimize photobleaching and phototoxicity; collectively, these features allow for long-term imaging (Ahrens et al. 2013; Huisken and Stainier 2009; Keller et al. 2008; Krzic et al. 2012; Mertz 2011; Pampaloni et al. 2007; Planchon et al. 2011; Truong et al. 2011; Wan et al. 2019).

Remarkably, LLSM using an ultrathin light sheet (Fig. 1a, right and 1b, right) enables whole-cell 3D live imaging at unprecedented spatiotemporal resolution for extended periods of time. This is achieved by scans of cellular 3D volumes at subsecond intervals at a resolution of ~230 nm laterally and ~ 370 nm axially (Chen et al. 2014). In contrast to the conventional light sheet created by several micron-thick Gaussian beams (Fig. 1b, left), the lattice light sheet is generated by a massive parallel array of non-diffracting light beams (Bessel beams) that mutually interfere to create an ultrathin light sheet ~0.4–1 μm-thick extending over cellular dimensions (Fig. 1b, right) (Chen et al. 2014). A Bessel beam is a special class of non-diffracting beam that allows a narrow beam width to propagate without spreading over a long distance due to the self-interference effect of the beam (Fig. 1b, middle) (Durnin 1987; Durnin et al. 1987; Planchon et al. 2011). Scanning with an ultrathin light sheet minimizes fluorescence photobleaching and phototoxicity, permitting acquisition of high-resolution images of whole cells for hundreds of volumes (Chen et al. 2014; Gao et al. 2012; Planchon et al. 2011). In the original LLSM model, the excitation beams are illuminated diagonally through a water-dipping objective lens (Fig. 1a, right) to place it in close proximity to specimens. However, a model with an inverted type configuration using specialized lenses has recently been commercialized.

Another important advantage of LLSM is that, unlike conventional super-resolution microscopy techniques such as stimulated emission depletion (STED) microscopy and photoactivated localization microscopy (PALM), it does not require the use of special fluorescent dyes or proteins. STED microscopy functions by depleting fluorescence surrounding the excitation focal spot while leaving a center spot active to emit fluorescence; thus, appropriate choices of dyes and lasers are critical to maximize performance (Dyba et al. 2003). For PALM, photo-switchable dyes or fluorescent proteins are required to localize individual fluorophores by repeating the process of photoactivation, measurement, and bleaching of isolated single molecules over numerous cycles (Betzig et al. 2006). Therefore, dye-independent LLSM has a wide range of applications including highly flexible multicolor imaging (Valm et al. 2017), fluorescence resonance energy transfer imaging (O'Shaughnessy et al. 2019), expansion microscopy (Gao et al. 2019), and use of existing various transgenic animal models.

## Image collection by LLSM

LLSM offers two imaging modes: super-resolution structured illumination microscopy (SIM) and high-speed dithered (Chen et al. 2014; Li et al. 2015). In SIM mode, more than 200 3D volumes can be acquired at 4-s intervals at a resolution of 150 × 280 nm *xz*, and multiple images are collected at each *z*-plane. In dithered mode, the two-dimensional (2D) lattice pattern is oscillated using a galvanometer to provide time-averaged uniform illumination, and only one 2D image at each *z*-plane is acquired at a rate of up to 100–200 frames per second and resolution of 230 nm in *x* and ∼370 nm in *z*. This is ∼1.3–1.5 times poorer in each direction than SIM mode, but dithered lattices achieve about 7.5-times faster imaging at a comparable signal-to-noise ratio. Furthermore, use of dithered mode to collect single-plane images at each *z*-plane minimizes photobleaching and phototoxicity, enabling data collection for thousands of time points. Overall, the dithered lattice light sheet is advantageous for most biological phenomena, including mitosis, unless additional resolution is required.

A more recent LLSM model with adaptive optics (AO), which correct for sample-induced aberrations in multicellular specimens (Booth 2007; Ji et al. 2010; Royer et al. 2016; Wang et al. 2014), was used to visualize mitotic processes in large multicellular volumes, such as organoids and Zebrafish embryos (Liu et al. 2018). Spatiotemporal resolution and non-invasiveness of this system are comparable to the original LLSM model, but tiled acquisition collects a larger volume (e.g., > 200 × 200 × 100 μm), thus covering a wide range of developing tissues (Fig. 1c, left). To visualize individual cells within the crowded multicellular environment of the intact organism, the plasma membrane staining was used to computationally separate each cell in a virtual space and isolate any desired cell (Fig. 1c, right).

The ability of LLSM technology to generate large amounts of image data (easily reaching 100 GB to 1 TB) for each time lapse along with thousands of numerical datasets results in far more information than can be analyzed by conventional approaches. This newly emerging “big image data” problem is a critical challenge that must immediately be resolved, as will be discussed in a later section.

## Imaging of mitotic processes with LLSM

**Fig. 2:**
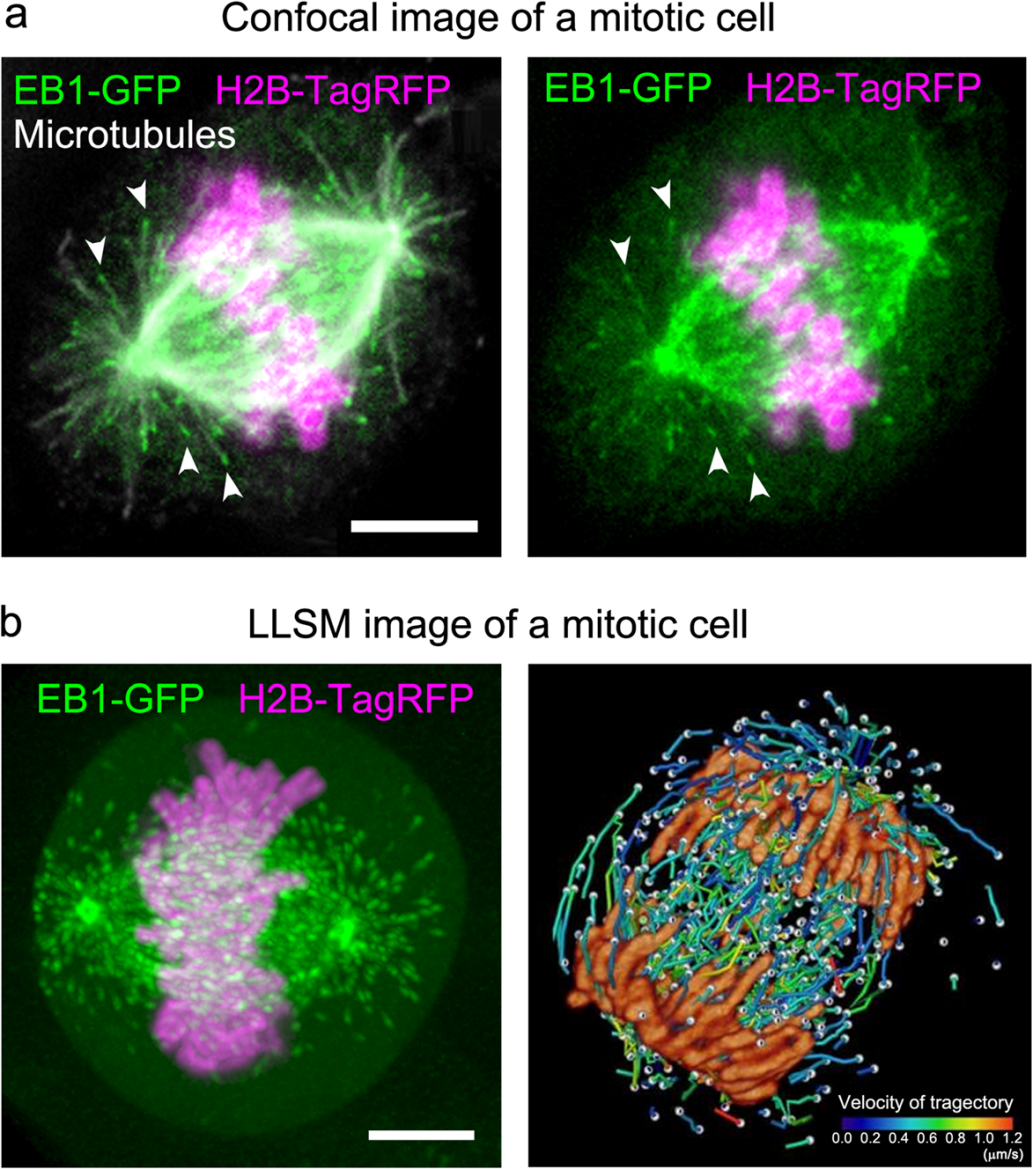
Comparison of 3D stack images collected by conventional confocal microscopy and LLSM. **a** Distribution of EB1-GFP in a mitotic HeLa cell at metaphase. HeLa cells (clone A1) stably expressing EB1-GFP (green) and H2B-TagRFP (magenta) (Chen et al. 2014) were fixed and immunostained for microtubules (white) to identify the positions of microtubule ends. Z-stack images were collected under a conventional laser-scanning confocal microscope. Scale bar: 5 μm. Data is reused from Ref. Yamashita et al. 2015. **b** Images collected by LLSM (left) were displayed in a 3D space using Imaris software. Note that individual EB1 comets can be discriminated even inside a thick spindle. Scale bars: 5 μm. Data is reused from Ref. Yamashita et al. 2015. On the right, trajectories of EB1-GFP movement are shown as balls and lines. The colored bar indicates the range of the mean travel speed of EB1-GFP comet trajectories (0.0–1.2 μm/s). Adapted from Ref. Chen et al. 2014

The remarkable improvement in axial resolution of LLSM provides, for the first time, a complete overview of whole mitotic cells as a time-lapse video with submicron 3D resolution (Fig. 2). Importantly, this information can be observed from any viewing angle with similar resolving power by simply rotating the image (Chen et al. 2014; Liu et al. 2018; Yamashita et al. 2015; Yamashita et al. 2020). Such 3D representations of data clearly demonstrate the advantages of LLSM over conventional laser confocal methods (Fig. 2). In this section, examples of mitotic processes visualized with LLSM over a wide range of scale are introduced—from single cultured cells to cells constituting developing tissues.

### Remodeling of organelles during mitosis

At the onset of mitosis, every membrane-bordered organelle, including mitochondria, Golgi apparatus, and endoplasmic reticulum (ER) including the nuclear envelope, undergo vast rearrangements to be accurately inherited and delivered to their proper locations in daughter cells (Warren and Wickner 1996). This process has fascinated cell biologists since the early 1900s, when specific cytochemical markers capable of labeling individual organelles were first developed for light microscopy (Wilson 1925). Although determining how theses organelles transform their architecture in detail remains of great interest, such studies are challenging because of their complex 3D morphology and the rapidity of changes in cell organization as cell division progresses.

**Fig. 3:**
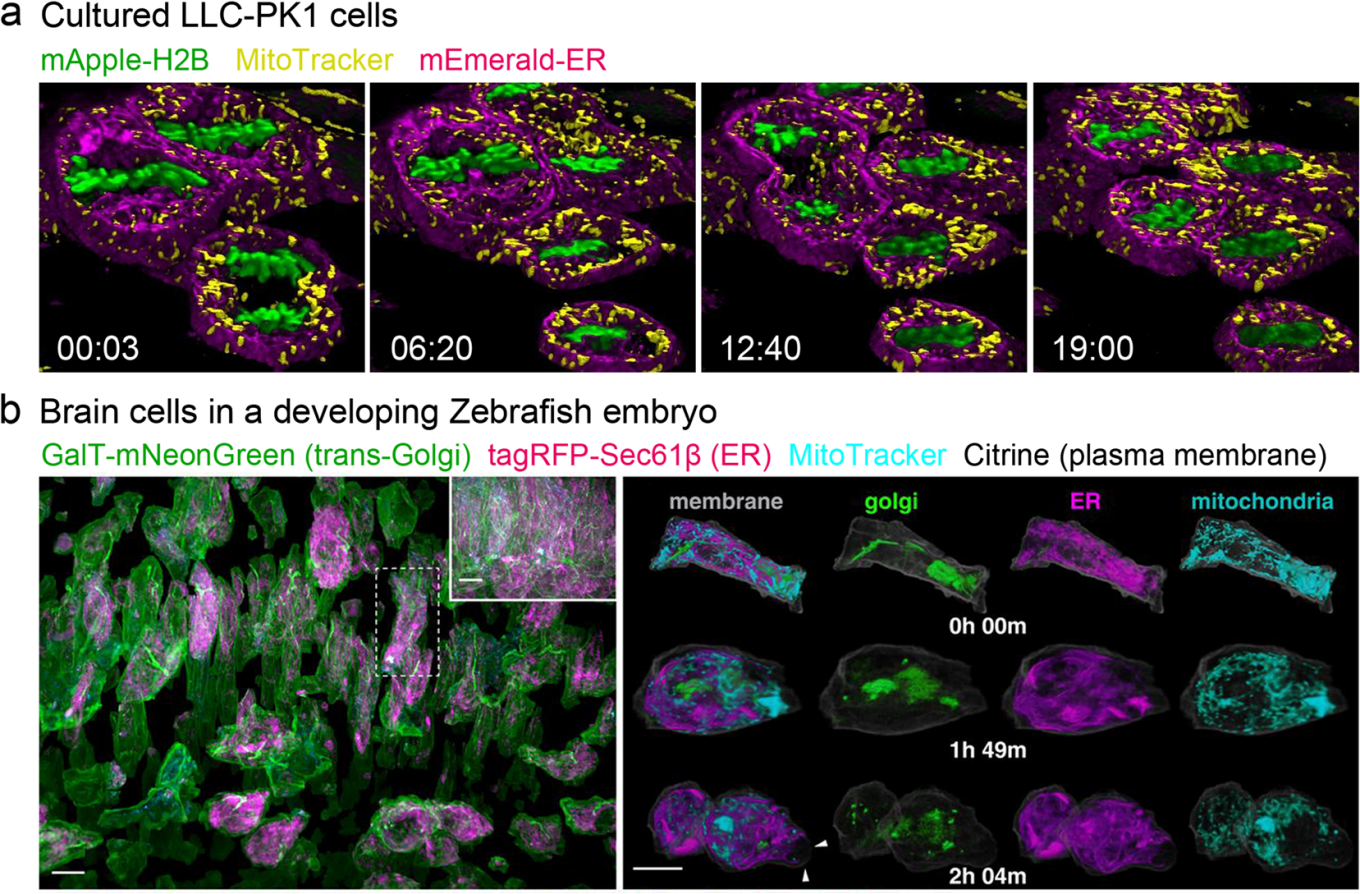
Reorganization of organelles during mitosis visualized with LLSM. **a** Changing morphologies of histones (green), mitochondria (yellow), and ER (magenta) in dividing cultured LLC-PK1 cells. Four time points in a slab extracted from a larger 3D time-series dataset imaged for 300 time points at 3.8-s intervals are shown. Adapted from Ref. Chen et al. 2014. Time scale: m:s. **b** Changing morphologies of trans-Golgi (green), ER (magenta), mitochondria (cyan), and plasma membrane (gray) in the brain of a developing Zebrafish embryo. Left: Computationally separated neural progenitor cells from a 70 × 35 × 35-μm region (inset) of a brain. Right: Changing morphologies of the organelles in the specific cell boxed in (a) at three time points through mitosis. Arrowheads indicate mitotic blebs. Scale bar, 10 μm. Adapted from Ref. Liu et al. 2018 under the Copyright (5004591427363)

Chen et al. labeled LLC-PK1 cells by stable transfection with mEmerald-ER and mApple-H2B (histone H2B), stained them with MitoTracker Deep Red dye, and recorded 300 3D volumes at 3.8-s intervals using LLSM (Chen et al. 2014) (Fig. 3a). As previously observed, mitochondria divided during mitosis (Taguchi et al. 2007) and the ER reorganized into large, extended cisternae, such that little remained of the reticular network observed during interphase (Lu et al. 2009). Additionally, LLSM revealed that mitochondrial fragments are corralled by the ER cisternae as they move within and between these pockets. However, exchange of fluorophore between mitochondria and the ER, suggesting their mutual fusion, was not observed.

AO-LLSM was used to study various 3D subcellular processes throughout the cell cycle across populations of cells in vivo (Liu et al. 2018). To simultaneously examine the dynamics of multiple organelles throughout the cell cycle across a population of cells in Zebrafish embryos, brain progenitor cells with markers for trans-Golgi, ER, mitochondria, and plasma membrane were imaged (Fig. 3b). To visualize individual cells, the cells were computationally separated and desired cells were isolated. In interphase, multiple trans-Golgi segments were observed, which often appear as long filaments preferentially aligned along the axis of cell polarization (Fig. 3b, left) that fragmented during mitosis (Fig. 3b, right). The ER formed a reticular network in interphase and sheet-like cisternae during mitosis, as observed in cultured cells. Mitochondria formed punctate structures near the surface and longer tubules in the subset of more deeply buried interphase cells (Fig. 3b, right). Mitochondria were preferentially located nearer the plasma membranes during mitosis, whereas other organelles were distributed uniformly.

### 3D tracking of spindle microtubule growth

Direct observation of individual filaments in the mitotic apparatus, which comprises hundreds of microtubule filaments (Inoué and Sato 1967; Inoué 1981; Salmon 1975), has never been achieved (Mitchison et al. 1986; Waterman-Storer et al. 1998) because of thickness of these structures and the high density of labeled protein. Recently, we detected microtubule growth dynamics in 3D at the whole-cell level using LLSM (Chen et al. 2014; Yamashita et al. 2015) in conjunction with a GFP-conjugated form of the microtubule growth marker protein end-binding 1 (EB1-GFP), a microtubule plus-end-tracking protein (Mimori-Kiyosue et al. 2000). EB1 binds selectively to the growing ends of microtubules in a comet-shape pattern with a short axis diameter of approximately 25 nm and anteroposteriorly elongated tail of up to 500 nm (Mimori-Kiyosue et al. 2000; Morrison et al. 1998). Notably, the utility of EB1-GFP as an analytical tool to study microtubule growth has been demonstrated in living cells and animals (Abe et al. 2011; Matov et al. 2010; Muroyama and Lechler 2017; Srayko et al. 2005). Time-lapse imaging and tracking of EB1-GFP comets yields microtubule growth trajectories, which permit analysis of microtubule growth rates, localization, and directionality (Applegate et al. 2011). However, until recently, image acquisition and analysis were predominantly limited to a 2D plane because of insufficient spatiotemporal resolution of conventional fluorescence microscopes.

**Fig. 4:**
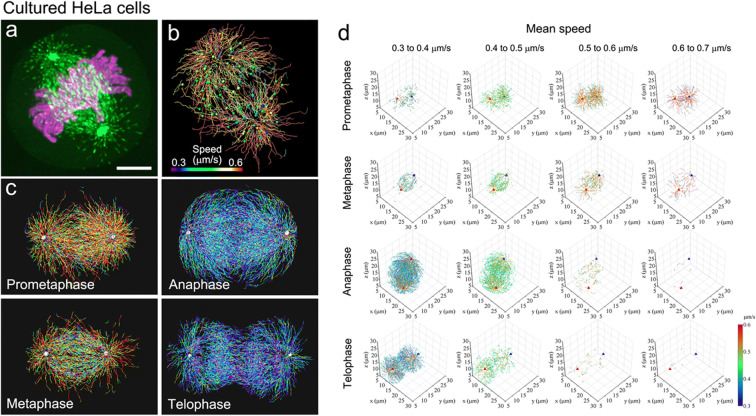
Analysis of spindle microtubule growth as digital spindles. **a** A representative image of a HeLa cell (clone A1) expressing EB1-GFP (green) and H2B-TagRFP (magenta) from a time-series 3D dataset collected with LLSM. **b** An example of tracking of EB1-GFP comet movement. Trajectories are shown with colors corresponding to the average speed of each trajectory and dots at the end of the trajectory. The colored bar indicates the mean speed (μm/s). **c** Representative images of EB1-GFP trajectories at different mitotic phases. Trajectories were generated from 75-frame sequences corrected at 0.755-s intervals. The color code is the same as shown in (b). **d** 3D distribution of EB1-GFP comet trajectories moving at different rates in each mitotic phase. Comet speed data were divided into 10 classes spanning the entire data range (0 to 1 μm/s) using custom tools created in Matlab (Yamashita et al. 2015). Data included in the 0.3–0.7 μm/s range are shown in 3D coordinates. Blue and red triangles indicate centrosomes. Color bar indicates the mean speed (μm/s). Data are from Ref. Yamashita et al. 2015

Using LLSM to generate a time-lapse sequence of mitotic HeLa cells expressing EB1-GFP and H2B-RFP at 0.755-s intervals over a 56.625-s duration (75 timepoints) resulted in the detection of >10,000 EB1-GFP comets and > 2000 trajectories for each mitotic cell (Yamashita et al. 2015) (Fig. 4a–c). To analyze tracking results obtained as a large number of time-series *x*-*y*-*z* coordinate datasets, we developed a data processing pipeline. Because the spindle apparatus often rotates and changes its orientation, changes in the angular orientation of the spindle were compensated using the centrosome positions as references to compare all datasets under a common reference frame. These data, “digital spindles” (Yamashita et al. 2020), can be used to generate an average image for each phase, classify trajectories by growth speed and position, and analyze growth speed and angles of individual microtubules in any area of interest (Fig. 4d). Such techniques are useful for precise phenotyping of cell division under different conditions, e.g., for comparison of normal and disease states (Kawasaki et al. 2020).

### Karyotyping in living cells

Identification and tracking of individual chromosomes in living cells will reveal new insights into chromosome arrangements and how they change between healthy and diseased conditions. However, this has proven difficult for conventional epifluorescence methods including confocal microscopy due to inadequate axial resolution, the rounded shape and thickness of mitotic cells, and their sensitivity to light (Khodjakov and Rieder 2006). It is, however, a problem that can be solved by the high-resolution, low photobleaching/phototoxicity, and reduced out-of-focus background of ultrathin light-sheet illumination.

**Fig. 5:**
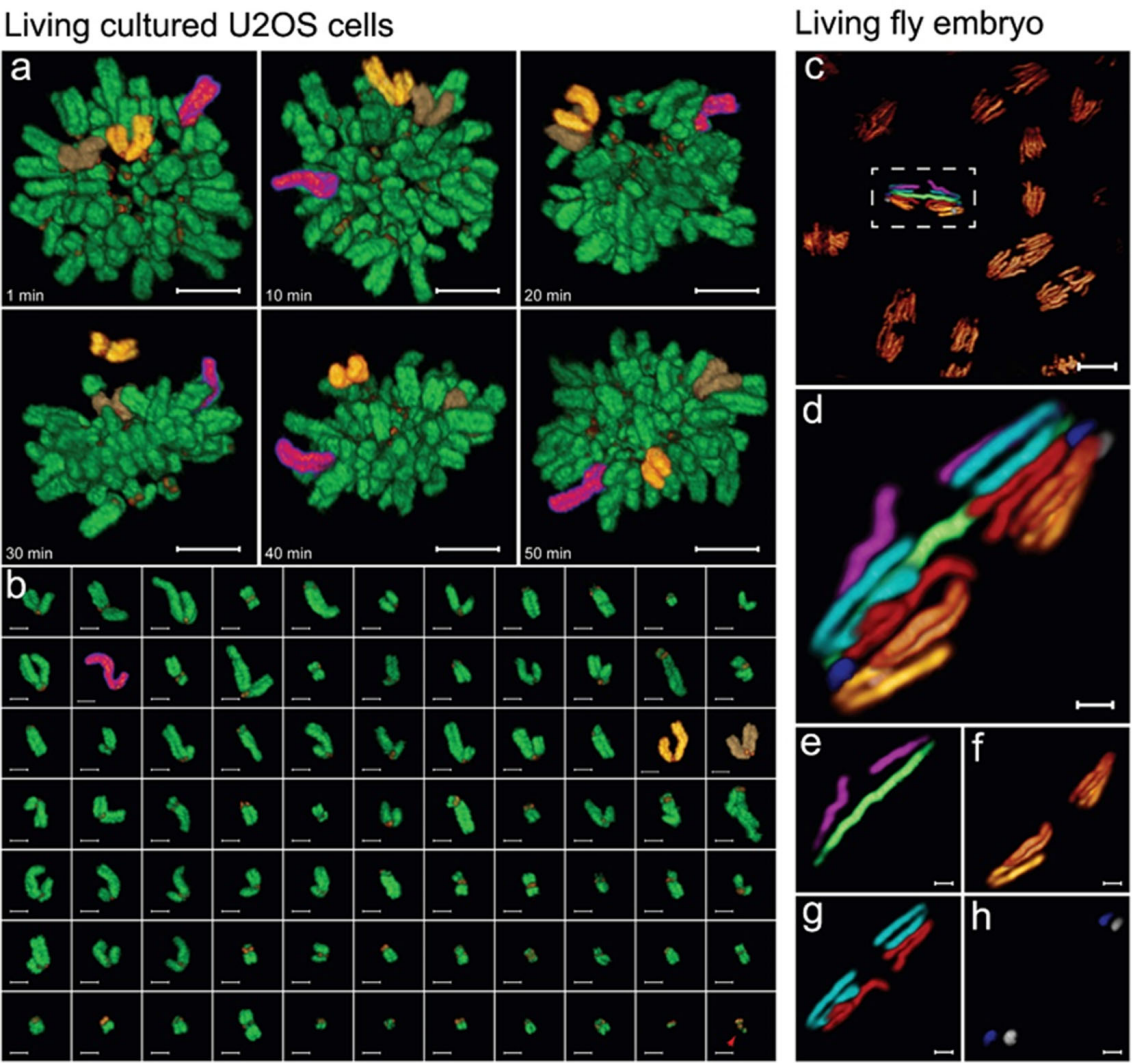
Identification of individual chromosomes in cells. **a, b** Karyotyping of living cultured U2OS cells. Dual-color imaging of chromosomes (green) and kinetochores (red) at six time points from prometaphase to metaphase, selected from a series of 50 volumes acquired at 60-s intervals and collected using Bessel-plane SR-SIM (a). Three specific chromosomes (colored magenta, brown, and yellow) were identified by shape throughout the observation period. Karyotyping by manual segmentation of all chromosomes at the initial time point (b). Chromosomes were identified using the position of kinetochore pairs. The cell exhibits extreme polyploidy, with 76 diploids and one possible triploid (red arrow). Scale bars: 5 μm in (a); 2 μm in (b). **c**, **d**, **e**, **f**, **g**, **h** Chromosomes in mitotic cells at the surface of a living syncytial embryo of *D. melanogaster*, visualized with Bessel beam plane SR-SIM (c). Enlarged view of the boxed chromosome in (c), in which individual chromosomes are differently colored (d). Sex chromosomes isolated from (d) identified the embryo as female (e), and autosomes 2, 3, and 4 could be identified (f–h). Adapted from Ref. Gao et al. 2012 under the Copyright (5004601453014).

Gao et al. used super-resolution structured-plane illumination (SR-SIM) with a periodic Bessel beam (Bessel-plane SR-SIM) to image living U2OS cells expressing mEmerald-H2B and tdTomato-CENP-B, a kinetochore marker (Gao et al. 2012) (Fig. 5a, b). To improve wide-field SR-SIM and its application in thick living specimens, SR-SIM combines the tightly confined planar illumination of a periodically stepped Bessel beam with the principles of wide-field 3D super-resolution structured illumination microscopy (wide-field SR-SIM) (Gustafsson et al. 2008). In cells examined with SR-SIM, movement of individual chromosomes to the metaphase plate was observed in 3D (Fig. 5a). In this massively polyploid cell, all 77 chromosomes could be identified at any time point by manual segmentation of all chromosomes (Fig. 5b). In each case, kinetochore pairs correctly locating at their corresponding chromatids were used to identify specific chromosomes.

Gao et al. also observed mitosis in multicellular systems using Bessel-plane SR-SIM. In a living syncytial embryo of *D. melanogaster*, chromosomes of multiple cells within 10–20 microns of the surface could be viewed with full-extended resolution (Fig. 5c). As with single-cultured cells, the karyotype could be determined; specifically, autosomes 2, 3, and 4 and sex chromosomes identified the embryo as female (Fig. 5d–h).

Combined with the accurate segmentation and shape information provided, this technique uniquely identifies and tracks each chromosome in living cells, allowing determination of chromosome abnormalities and their effect on cellular phenotypes. Notably, this example was based on Bessel beam microscopy, a prototype of LLSM; thus, the improvements achieved by LLSM should yield similar or more accurate results, especially with LLSM SIM mode.

### Chromosomal passenger in early *C. elegans* embryos

**Fig. 6:**
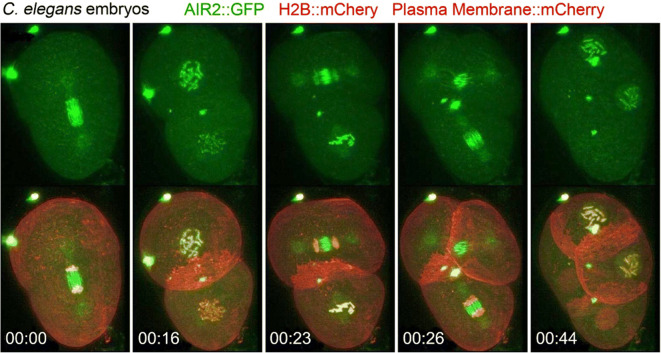
Localization of the chromosomal passenger protein AIR-2::GFP during cell divisions of the early *C. elegans* embryo. The thinness of the lattice light sheet allowed AIR-2::GFP distribution to be followed with remarkable detail. Time scale: h:m. Adapted from Ref. Chen et al. 2014

In early embryos of *C. elegans*, the cell cycle rapidly occurs by alternate repeating of S and M phases. An embryo expressing GFP-tagged Aurora B kinase homolog AIR-2, a chromosomal passenger complex protein (Carmena et al. 2012), was imaged with LLSM (Fig. 6). As previously reported, AIR-2::GFP mainly localized to metaphase chromosomes, followed by midbody microtubules in anaphase to telophase, and eventually in a persistent midbody remnant after cytokinesis (Schumacher et al. 1998). However, the thinness of the lattice light sheet allowed this process to be followed at a high signal-to-noise ratio with remarkable detail. AIR-2::GFP was first detected on chromosomes in prophase. By anaphase, it was most concentrated at spindle midzone microtubules, as previously observed, although some remained at the chromosomes and centrosomes, and faint signals were observed also along astral microtubules. High-spatiotemporal analysis of previously undetected distributions of mitosis regulators may provide new insight into how these molecules regulate cell division in developing embryos.

### Morphology of dividing cells in different organs of developing Zebrafish embryos

**Fig. 7:**
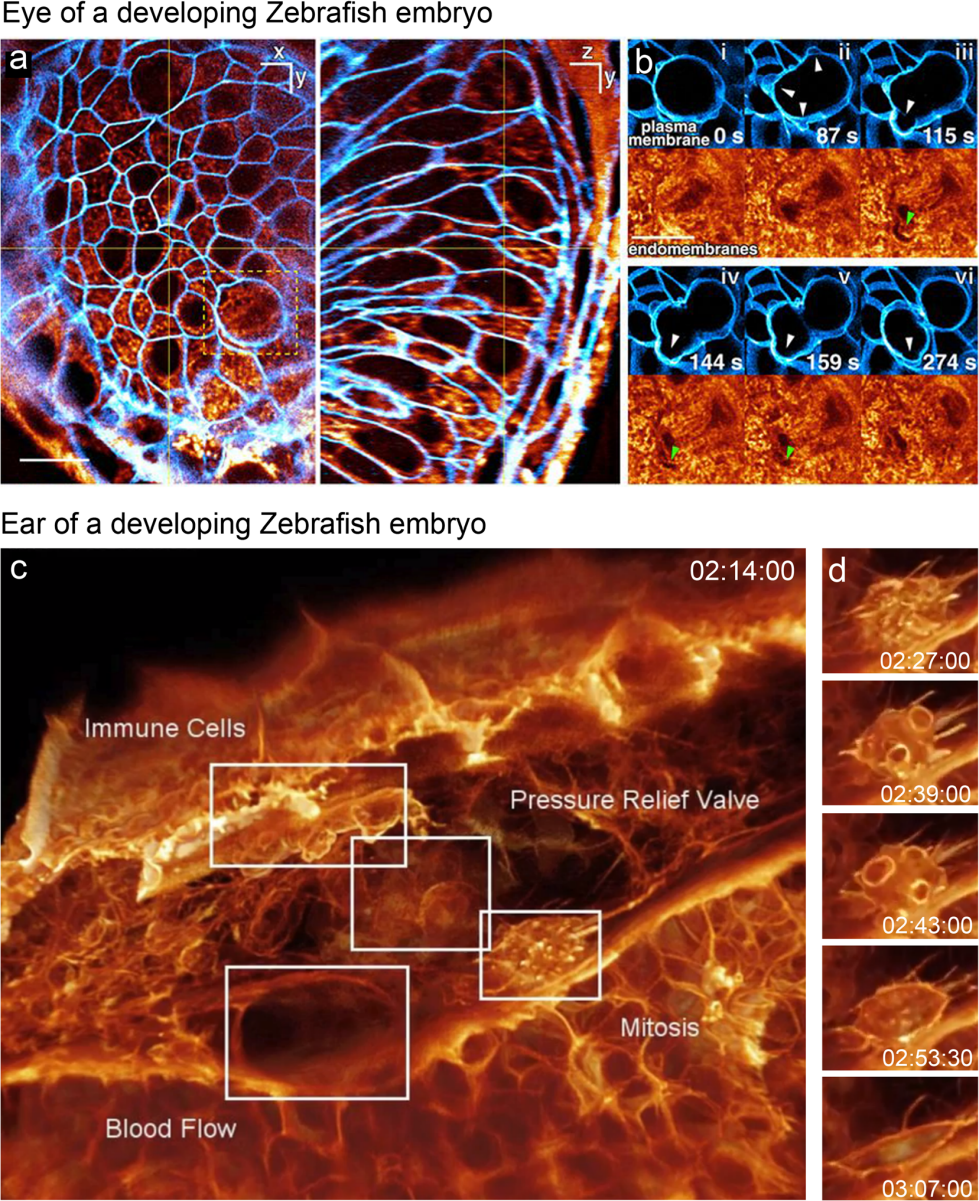
Changing morphologies of the cell surface during mitosis *in vivo* visualized with LLSM. **a**, **b** Membranes in the developing Zebrafish eye from a time-series 3D video collected with AO-LLSM at 43.8-s intervals for 200 time points. Plasma membrane visualized with membrane-citrins (blue) and endomembrane stained with Bodipy–tetramethylrhodamine (TMR) (orange), viewed as 1-μm-thick slab orthoslices (a). Six time points from the video showing plasma membrane blebs (white arrowheads) during mitosis and the exclusion of endomembranes in early blebs (green arrowheads). **c**, **d** A mitotic cell within the perilymphatic space next to the inner ear of a living transgenic Zebrafish embryo expressing membrane-Citrine. A 3D view of the tissue from a time-series 3D video collected for 438 time points at 13-s intervals (a). Cells and structures observed are indicated. The morphology of mitotic cells at different five different time points (d), indicating production of transient blebs. Time scale: h:m:s. Adapted from Ref. Liu et al. 2018 under the Copyright (5004591427363)

Observation of different organs in Zebrafish embryos using AO-LLSM revealed phenotypic diversity across different cell types and developmental stages, as well as similarities and differences in mitosis progression, compared with cultured cells (Liu et al. 2018). The results revealed changes in the organization and position of organelles as cell division progresses, including the volume of cells and contained organelles. For example, formation of plasma membrane blebs observed transiently before cytokinesis in culture (Boss 1955; Laster and Mackenzie 1996) was commonly observed in various Zebrafish organs (Fig. 7). In contrast, although it has been shown that the shape and total surface of a cell change during mitosis in cultured cells (Aguet et al. 2016; Boucrot and Kirchhausen 2007), the total cellular volume remained constant throughout mitosis in the eye and ear observed with AO-LLSM (Fig. 7b).

## Big image data problem

As mentioned above, LLSM technology provides far more information than can be analyzed by conventional approaches that depend on human eyes and manual editing. Efforts to tackle this newly emerged challenge in cell biology have just begun. Image analysis that requires manual editing of series of images containing high-resolution spatiotemporal information is very time consuming and data processing for a pipeline requires extensive calculations, making it difficult to routinely process a large number of datasets in typical biology laboratories. Therefore, development of automated and user-friendly computational tools, and their adaptation for widespread use across biological research fields are essential for practical use of LLSM technology.

Recently, a method to generate a dynamic protein atlas of human cell division was reported (Cai et al. 2018). The authors automatically imaged mitotic cells in culture using a conventional confocal microscope, and then calibrated the signal by fluorescence-correlation spectroscopy to convert protein fluorescence images to time-resolved distribution maps of protein concentrations. Using these data, they generated a canonical model of morphological changes of cells as mitosis progresses. Such an approach to time-series 3D volume data provides new opportunities for big image data mining.

In information science, one of the most successful advances in big data analysis in recent years has been deep-learning technologies (LeCun et al. 2015; Min et al. 2017; Shen et al. 2017). Ronneberger’s group reported U-Net, a generic deep-learning technique for quantification tasks in biomedical image data, such as cell detection and shape measurements (Falk et al. 2019; Ronneberger et al. 2015). U-Net featuring pretrained models for single-cell segmentation can work on ImageJ software (Schneider et al. 2012), the most popular image analysis tool, thus enabling non-machine-learning experts to analyze data with only a few annotated samples as training data. However, the inspection and quantification of 3D images remains difficult, and tools for subcellular 3D morphometry have not been developed (Driscoll and Danuser 2015). Recently, a generic morphological-motif detector for 3D images capable of automatically identifying lamellipodia, filopodia, blebs, and other motifs using u-shape3D (a computer graphics and machine-learning pipeline) was reported (Driscoll et al. 2019). Extending this technology to analyze mitotic cells is a promising direction.

## Conclusions

Recent innovations in high-resolution live-cell 3D microscopy using LLSM technology have begun to enable imaging of mitotic processes with unprecedented detail. This imaging technique provides a rich source of cellular information that far exceeds the information that cell biology has gained thus far. However, at present, no standardized methodologies for big image data analysis exist. Thus, incorporation of the latest information science technology to analysis of images and data extracted from the images is a critical and urgent challenge. Once a functional environment for big image data analysis has been established, this comprehensive measurement and analysis system will be a powerful tool for exploring novel molecular mechanisms regulating mitosis in normal and disease states, as well as high-throughput, high-content screening for precise and accurate drug discovery.

## Abbreviations

LLSM: Lattice light-sheet microscopy
GFP: Green fluorescent protein
EB1: End-binding1
SR-SIM: Super-resolution structured illumination microscopy
AO: Adaptive optics
ER: Endoplasmic reticulum
STED: Stimulated emission depletion
PALM: Photoactivated localization microscopy
